# Constipation and Parkinson disease: A 2-sample bidirectional Mendelian randomization analysis

**DOI:** 10.1097/MD.0000000000043240

**Published:** 2025-08-22

**Authors:** Huaxing Li, Guolan Su, Weilan Kang, Weihao Huang, Aiwu Liu, Meihui Zhang

**Affiliations:** a Department of Neurology, Guangdong Tongjiang Hospital, Foshan, Guangdong Province, China.

**Keywords:** constipation, genetics, Mendelian randomization, Parkinson disease

## Abstract

Patients with Parkinson disease (PD) have a higher risk of having constipation and vice versa. But so far it is not clear whether there is a causal relationship. Therefore, a 2-sample bidirectional Mendelian randomization study was performed to investigate the potential bidirectional association between constipation and PD. Independent genetic variants strongly associated with constipation were obtained from the FinnGen consortium. Data for PD were collected from the genome-wide association study summary data. We explored the causal relationship between constipation and PD using publicly available genome-wide association study data. Analysis of constipation’s effect on PD identified 16 significant single nucleotide polymorphisms, all with strong instrumental validity. Genetic susceptibility analysis did not suggest any statistical significance between constipation and PD (odds ratio = 0.77, 95% confidence interval 0.57–1.04; *P* = .097). In the analysis of PD’s impact on constipation, 20 significant single nucleotide polymorphisms were identified; however, genetic susceptibility analyses found no causal effect (odds ratio = 1.00, 95% confidence interval 0.97–1.04; *P* = .845). Our bidirectional Mendelian randomization analysis revealed no significant genetic association between constipation and PD in European populations, challenging the prevailing hypothesis of constipation as an early prodromal feature of PD. These null findings persisted across both forward and reverse causal directions. However, we cannot exclude the possibility of residual confounding through shared genetic architectures or undetected environmental covariables. Future studies incorporating larger multiethnic cohorts and comprehensive covariate adjustment are warranted to elucidate this association.

## 1. Introduction

Parkinson disease (PD) is a neurodegenerative disease pathologically defined by Lewy body inclusions in the brain and the death of dopaminergic neurons in the midbrain.^[1]^ Its clinical manifestations are mainly resting tremors, rigidity, bradykinesia, and postural instability.^[2]^ Also common are non-motor symptoms such as loss of smell, sleep disorders, and gastrointestinal symptoms, particularly constipation.^[3]^ About 79% of PD patients exhibit prolonged colonic transit time,^[4]^ and the same is probably true for de novo PD patients.

In addition, constipation is among the first non-motor symptoms to develop in the prodromal phase of PD.^[5]^ Emerging evidence highlights a dynamic, bidirectional signaling network between the gut, its microbial communities, and the brain (commonly termed the microbiota–gut–brain axis).^[6]^ Pathological α-synuclein deposition is present throughout the gastrointestinal tract up to 20 years preceding diagnosis of PD.^[7–9]^ Some studies have indicated that constipation is probably associated with PD.^[10,11]^ Research into how the gut–brain axis influences PD may reveal insights into disease etiology. Braak hypothesis posits that aberrant α-Syn accumulation initiates in the gut and propagates via the vagus nerve to the brain in a prion-like fashion.^[12]^ This notion is supported by pathophysiologic evidence: α-Syn inclusions appear early in the enteric nervous system and the glossopharyngeal and vagal nerves^[13]^ while vagotomized individuals are at reduced risk for PD.^[14]^ Further, injection of α-Syn fibrils into the gut tissue of healthy rodents is sufficient to induce pathology within the vagus nerve and brainstem.^[15]^ However, the notion that α-Syn aggregation initiates in the ENS and spreads to the CNS via retrograde transmission remains controversial^[16]^ and experimental support for a gut microbial connection to PD is lacking.

Conventional observational studies are prone to many biases inherent in observational designs, such as confounding factors and reverse causality biases.^[17,18]^ Randomized controlled trials are considered the gold standard for making causal inferences in health sciences. However, conducting randomized controlled trials to investigate this relationship would be prohibitively time-consuming and labor-intensive. The exact nature of constipation’s effect on PD risk remains incompletely understood, as conventional observational studies cannot fully disentangle potential biases from shared pleiotropic effects or reverse causation. Mendelian randomization (MR) has emerged as a powerful methodological approach to establish causal relationships between exposures and outcomes.^[19]^ Leveraging the natural randomization of genetic variants during meiosis, MR utilizes exposure-associated genetic polymorphisms as instrumental variables (IVs) to infer causal links between risk factors (e.g., constipation) and disease outcomes (e.g., PD). Since genetic variants are randomly assigned at conception and precede disease onset, MR analyses are inherently resistant to confounding and reverse causation, thereby providing more robust causal inference. In this investigation, we conducted a comprehensive bidirectional 2-sample MR analysis using large-scale genome-wide association study (GWAS) datasets to rigorously evaluate the potential causal relationship between constipation and PD, including its directionality and effect magnitude.

## 2. Materials and methods

**Figure 1.**
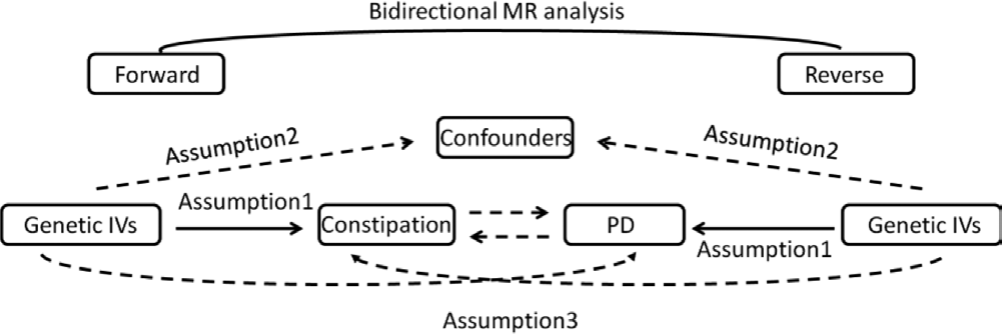
Flow chart of Mendelian stochastic analysis. The study satisfies the 3 main assumptions of Mendelian randomization: assumption 1: The solid line indicates that the instrumental variants directly affects the incidence of exposure. Assumption 2: dashed lines indicate that instrumental variables are not related to any potential confounders. Assumption 3: the instrumental variables affect the outcome only through the exposure, and not through any other causal pathways.

To investigate the causal link between constipation and PD, we used a 2-sample bidirectional MR research. Figure 1 depicts the research procedure.

### 2.1. Study design

We conducted a 2-sample MR study using data from the publicly available FinnGen (https://www.finngen.fi/en).https://storage.googleapis.com/finngen-public-data-r9/summary_stats/finngen_R9_K11_CONSTIPATION.gz. and the GWAS summary data (https://gwas.mrcieu.ac.uk/). https://gwas.mrcieu.ac.uk/datasets/ PD. All included study data were publicly available from Medical Research Council Integrative Epidemiology Unit, and FinnGen consortium, were approved by the relevant ethical review boards and participants gave informed consent, therefore no further ethical review was required.

This MR analysis relied on 3 core instrumental variable assumptions: the genetic variants must be robustly associated with the exposure (relevance assumption), not associated with potential confounders (exchangeability assumption), and affect the outcome only through the exposure (exclusion restriction criterion).

### 2.2. Data sources

See Table 1.

**Table 1:** Details of traits used in Mendelian randomization analyses.

Trait	Consortium	Population	Sample size	n_cases	n_controls
Constipation	FinnGen	European	309,154	26,919	282,235
Parkinson disease	IEU OpenGWAS project	European	482,730	33,674	449,056

GWAS = genome-wide association study.

### 2.3. MR of constipation on risk of PD

First, we identified 3 single-nucleotide polymorphisms (SNPs) strongly associated with constipation (*P* < 5 × 10^‐8^). Then, a more relaxed threshold (*P* < 5 × 10^‐6^) was used to identify SNPs due to the number of SNPs meeting genome-wide significance was only one (rs1983785). This helps to capture more genetic variations that may be associated with constipation, although it may reduce the false negative rate and increase the false positive rate.^[20–23]^ Second, to ensure genetic independence, we selected SNPs meeting stringent linkage disequilibrium criteria (*r*² < 0.001 and physical distance > 10,000 kb) based on European reference data from the 1000 genomes project.^[24]^ Third, in genetic association studies, 2 principal formulas are conventionally employed for calculating *F*-statistics: *F* = (N ‐ *k* ‐ 1)/*k* × *R*^2^/(1 − *R*^2^)^[25]^ and *F* = β^2^/SE^2^.^[26]^ The total *F* is calculated as: *F* = (N ‐ *k* ‐ 1)/*k* × *R*^2^/(1 − *R*^2^), and the *F*-statistics to test the strength of each instrument with the following formula: *F* = β^2^/SE^2^ (β, effect size [exposure]; SE, standard error [exposure]); *k* represents number of IVs. *R*^2^ is calculated as *R*^2^ = 2 × EAF × (1 − EAF) × β^2^, where EAF represents effect allele frequency, the *R*² value quantifies the proportion of phenotypic variance in constipation explained by each individual SNP, calculated using the previously described methodology. To minimize weak instrument bias, we applied a stringent threshold of *F*-statistic > 10 for SNP selection, ensuring robust instrument strength for subsequent analyses. Fourth, we systematically screened all candidate SNPs in both the IEU OpenGWAS database (gwas.mrcieu.ac.uk) and the GWAS Catalog (https://www.ebi.ac.uk/gwas/) to evaluate potential horizontal pleiotropy. Using a lenient significance threshold (*P* < 1 × 10^‐5^), we assessed whether these SNPs showed secondary associations with known PD risk factors or other phenotypes that could indicate pleiotropic effects. Established risk factors for PD encompass both genetic and environmental determinants. Key genetic contributors include mutations in SNCA, Parkin, DJ-1, PINK1, LRRK2, and VPS35,^[27]^ significant environmental exposures comprise pesticide exposure,^[28]^ mitochondrial dysfunction,^[29]^ and neurotoxic agents such as carbon monoxide, cyanide, carbon disulfide, and manganese poisoning. Additional risk factors include illicit drug use.^[30]^ For analyses, palindromic SNPs (i.e., SNPs whose alleles consist of a base and its complementary base) were excluded. In order to maintain consistency in SNPs used as IVs, across different analysis, we only used variants available for all examined traits and did not replace missing variants by proxies. To comprehensively evaluate pleiotropy and verify result robustness, we conducted Cochran *Q* test for heterogeneity assessment, MR-Egger intercept analysis (testing directional pleiotropy through regression intercept deviation), Mendelian Randomization Pleiotropy RESidual Sum and Outlier test (MR-PRESSO) for outlier detection and correction, Steiger directionality testing (ensuring correct exposure-outcome causal direction), leave-one-out sensitivity analysis (sequentially excluding individual SNPs to identify influential variants), and funnel plot asymmetry assessment (visualizing potential small-study effects) (with this comprehensive analytical framework effectively minimizing potential biases introduced during variant harmonization while validating the robustness of our primary findings).

### 2.4. MR of PD on risk of constipation

Take the same approach, PD data were derived from IEU OpenGWAS project. We identified SNPs robustly associated with PD (*P* < 5 × 10^‐8^). We then screened these SNPs in both the IEU OpenGWAS database and GWAS Catalog for potential horizontal pleiotropy by testing their associations (*P* < 1 × 10^‐5^) with constipation or its known risk factors. Well-known risk factors for constipation included, but were not limited to demographic factors (age, gender, income, education, work status, and geography), lifestyle factors and behaviors (physical activity, smoking, fiber, fluid, alcohol, and coffee intakes), and numerous health-related factors (including medical conditions and medications).^[31]^

### 2.5. MR analyses

Three MR analytical methods were conducted to assess the causal effects of constipation on PD in this study to avoid the influence of potential pleiotropic effects of genetic variants. The primary MR analysis was conducted by the random-effects inverse-variance weighted (IVW) method, which combines the Wald ratio estimates of each SNP on the outcome to gain a pooled causal estimate and provides the highest statistical power. For random-effect IVW, it permits that all the instruments are ineffective on the condition that overall horizontal pleiotropy is balance. Furthermore, another 2 MR analyses, weighted median (WM) and MR-Egger, were implemented as complements to detect the causality. The WM method can generate unbiased causal estimates on the condition that at least 50% of the weight comes from valid IVs.^[32]^ The MR-Egger method provides consistent estimates accounting for pleiotropy on the condition that all the instruments are invalid, although with the lowest power. Moreover, the power to estimate a causal risk ratio of disease at a 2-side α of 0.05 and β of 80% was calculated with an online power calculation in MR studies with binary outcomes (https://shiny.cnsgenomics.com/mRnd/).^[23]^ And, we also calculate the minimum detectable odds ratios (ORs), with the following formula: ORmin = exp ((Z1 ‐ α/2 + Z1 ‐ β)/sqrt(N × *K* × (1 ‐ *K*) × *R*^2^)),^[23]^ where Z1 ‐ α/2 and Z1 ‐ β reflect the requirements of the significance level (α = 0.05) and statistical power (0.80), respectively; N, *K*, *R*^2^ represent the sample size, case proportion, and IV explanatory power, respectively.

### 2.6. Sensitivity analysis

Sensitivity analysis was conducted to detect the existence of horizontal pleiotropy, which violated the main MR assumptions. Thus, we perform Cochran *Q* test, MR-Egger intercept tests, MR-PRESSO, MR Steiger test, leave-one-out analyses, and funnel plot to examine the presence of pleiotropy to evaluate the robustness of the results. Specifically, the Cochran *Q* test was applied to evaluate the heterogeneity, which was detected if the *P* value was <.05. Horizontal pleiotropy was appraised by estimating the intercept term derived from MR-Egger regression, indicating potential bias with the intercept term difference from 0. MR Steiger test was applied to estimate the potential reverse causal association between PD and constipation. The leave-one-out analysis was performed to detect any pleiotropy driven by a single SNP.

All these MR analyses were performed using the TwoSampleMR package (version 0.5.1, University of Bristol, Bristol, United Kingdom) in R Version 4.3.2 (Posit, PBC, Boston).

## 3. Results

### 3.1. Causal effects of constipation on PD

We identified 16 independent SNPs significantly associated with constipation at genome-wide significance levels (*P* < 5 × 10^‐6^), meeting stringent linkage disequilibrium criteria (*r*² < 0.001 within 10,000 kb; Table S1, Supplemental Digital Content, https://links.lww.com/MD/P351). All instruments demonstrated robust strength (*F*-statistics > 10), effectively mitigating weak instrument bias. For the limited number of constipation-associated SNPs unavailable in the PD GWAS dataset, no appropriate proxy variants were identified. Following MR-PRESSO outlier testing (no outliers detected) and harmonization procedures (removing ambiguous/palindromic SNPs), these 16 high-quality SNPs were retained as final IVs (complete annotations in Table S1, Supplemental Digital Content, https://links.lww.com/MD/P351). In the present study, given a type I error of 5% and a statistical power of 0.80, the minimum detectable ORs for the PD is about 1.16, which means OR > 1.163 and OR < 0.837 have the same detection power.

**Figure 2.**
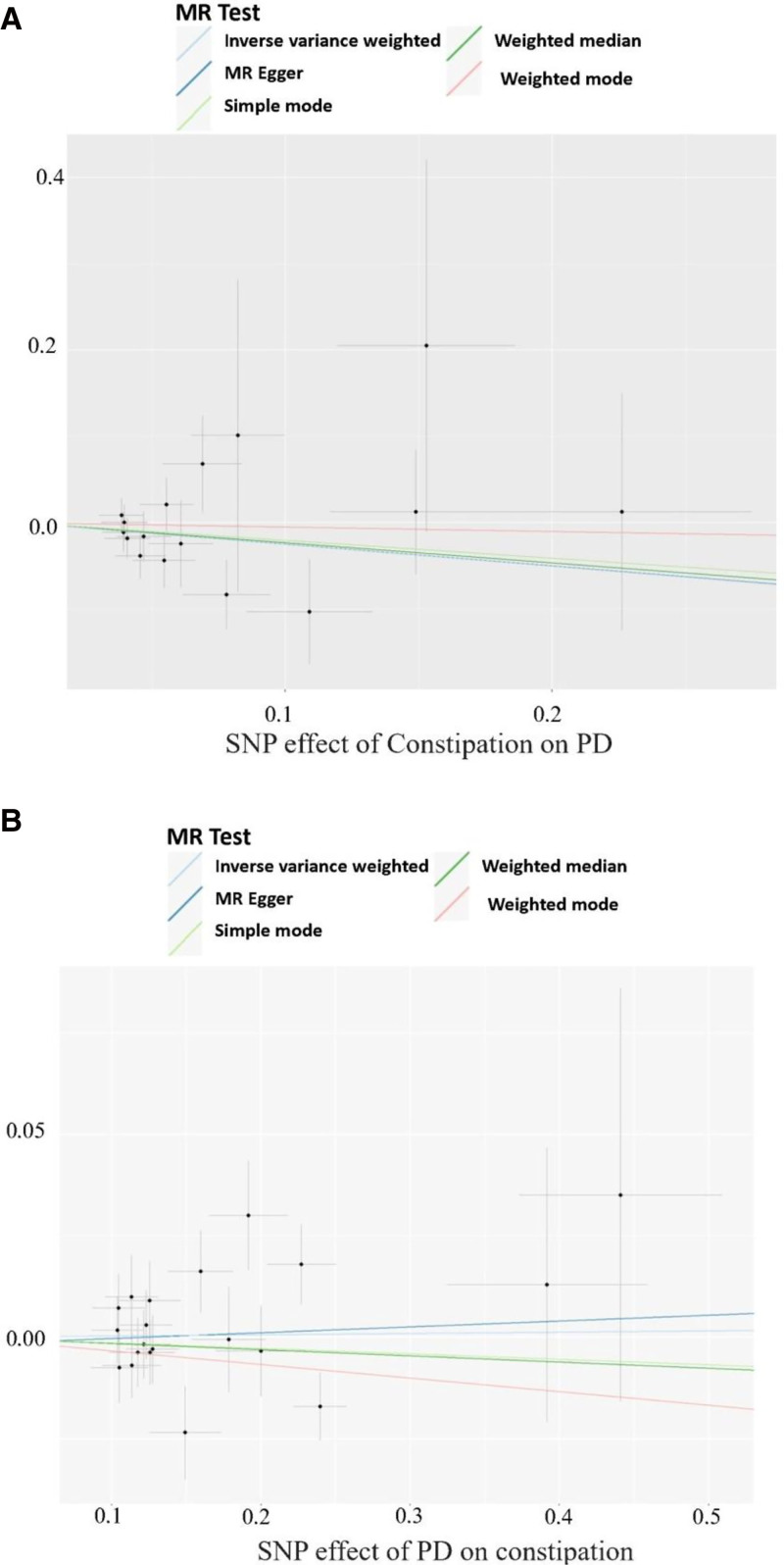
Scatter plots of causality. The slop of each line corresponds to the estimated MR effect in different models. (A) The MR study investigating the effect of constipation on PD; (B) the MR study investigating the effect of PD on constipation. PD = Parkinson disease, MR = Mendelian randomization.

The IVW method revealed no statistically significant association between genetically predicted constipation and PD risk (OR = 0.77, 95% confidence interval [CI] 0.57–1.04; *P* = .097). The power to estimate a causal risk ratio of disease is 0.97. The results from WM (*P* = .27) and MR-Egger (*P* = .53) analyses indicated statistically nonsignificant but directionally consistent effects (Fig. 2). To evaluate result robustness, we conducted sensitivity analyses using Cochran *Q* test, MR-PRESSO global test, MR Steiger directionality test, and MR-Egger intercept test (Table 2). *P* values was >.05 in the MR-PRESSO global tests and the MR-Egger intercept tests, manifesting that no horizontal pleiotropy existed across the analyses. MR Steiger test identified no evidence of reverse causality, and the causal direction was reliable. No significant heterogeneity was observed in the Cochran *Q* test analysis assessing the association between constipation and PD (*Q* = 13.37, *P* = .57).

**Table 2:** Mendelian randomization analysis results for the association between PD and constipation.

Method	Constipation on PD			PD on constipation		
	*B* (SE)	*P*-value	*Q* statistic/*P*-value	*B* (SE)	*P*-value	*Q* statistic/*P*-value
IVW	‐0.2526 (0.1526)	.09	13.36/.57	0.0032 (0.0165)	.84	24.43/.18
Weighted median	‐0.2340 (0.2122)	.27	–	‐0.0153 (0.0220)	.48	–
MR-Egger	‐0.2509 (0.3957)	.53	–	0.0144 (0.0544)	.79	–

IVW = inverse-variance weighted, PD = Parkinson disease, SE = standard error.

### 3.2. Causal effects of PD on constipation

**Figure 3.**
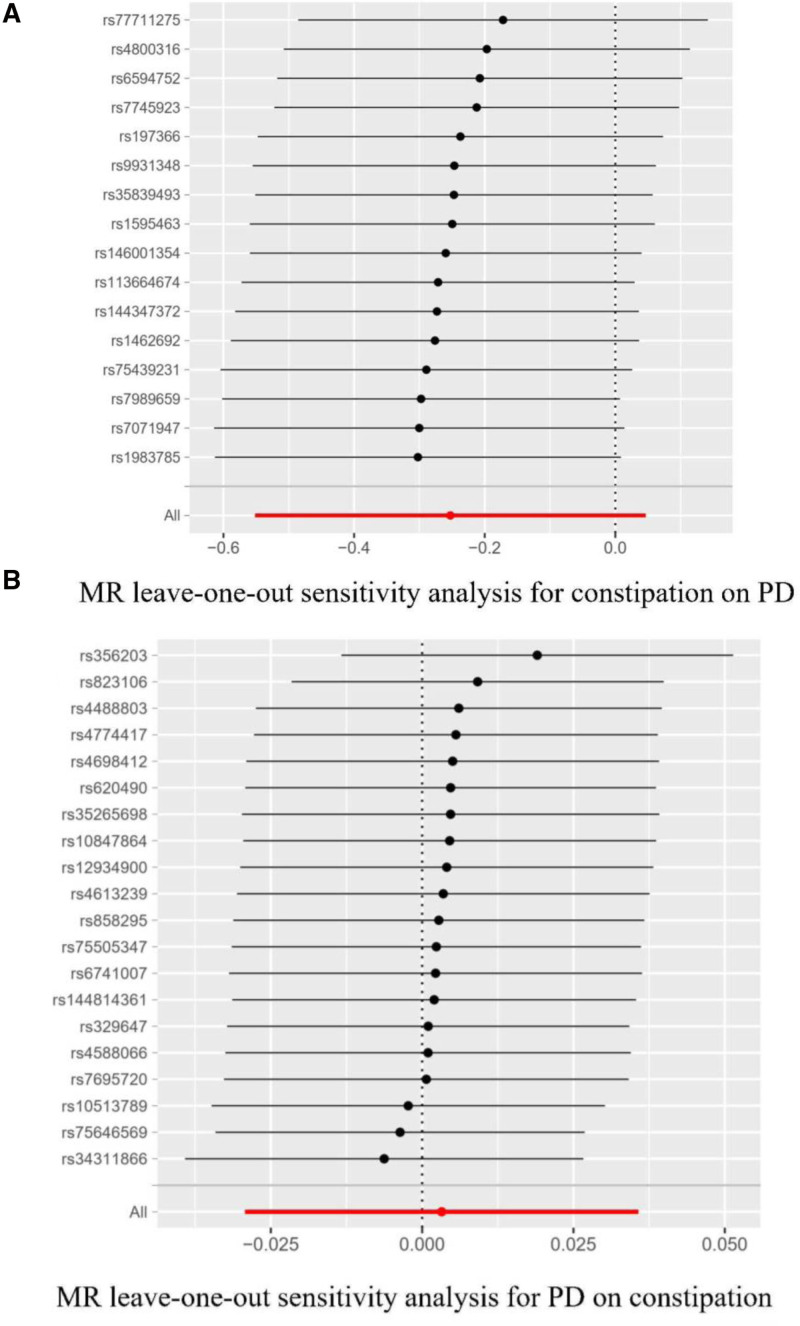
(A) Leave-one-out analysis for constipation on PD. (B) Leave-one-out analysis for PD on constipation. PD = Parkinson disease.

We identified 21 independent SNPs significantly associated with PD that satisfied the standard genome-wide significance threshold (*P* < 5 × 10^‐8^, *r*^2^ < 0.001, clumping distance = 10,000 kb) for exposure variables (Table S2, Supplemental Digital Content, https://links.lww.com/MD/P352). All instruments satisfied the strength assumption (*F*-statistics > 10), indicating no weak instrument bias in our MR analysis. No relevant proxy SNPs were identified to replace the small number of SNPs absent in PD GWAS data. Removing the following 1 SNP (rs10451230) for being palindromic with intermediate allele frequencies, 20 SNPs were selected as IVs (Fig. 3). Details of IVs of each constipation were exhibited in Table S2, Supplemental Digital Content, https://links.lww.com/MD/P352. In the present study, given a type I error of 5% and a statistical power of 0.80, the minimum detectable ORs for the constipation about 1.04 which means OR > 1.04 and OR < 0.96 have the same detection power. IVW analysis found no significant or suggestive evidence of an association between genetic liability to PD and the risk of constipation (OR = 1.00, 95% CI 0.97–1.04; *P* = .84). The power to estimate a causal risk ratio of disease is 0.06. The WM and MR-Egger methods yielded nonsignificant but directionally consistent estimates (Fig. 2). To assess robustness, sensitivity analyses were performed, including Cochran *Q* test, MR-PRESSO global test, MR Steiger test, and MR-Egger intercept test (Table 2). Both MR-PRESSO (*P* > .05) and MR-Egger intercept tests (*P* > .05) indicated no substantial horizontal pleiotropy. The MR Steiger test confirmed the absence of reverse causality, supporting the reliability of the causal direction. No heterogeneity was observed in Cochran *Q* test for the constipation-PD association (*Q* = 24.43, *P* = .18).

## 4. Discussion

Constipation is one of the most common gastrointestinal features of PD, occurring in over 50% of all PD patients during the course of their disease.^[33]^ Constipation is currently recognized as a risk factor of PD in relation to the number of evacuation per week and its severity.^[11]^ Furthermore, several studies have demonstrated that constipation may precede the occurrence of motor symptoms underlying an earlier involvement of the enteric nervous system and the dorsal motor nucleus of the vagus in the α-synuclein pathology.^[34]^ In PD, constipation is mainly due to slower colonic transit or puborectalis dyssynergia, but the concomitant use of antiparkinsonian, pain, and antidepressant medications may worsen it.^[35]^ However, current evidence regarding the constipation-PD association remains limited. While some studies have investigated this relationship, existing findings are restricted to observational correlations where reverse causality cannot be excluded. Leveraging large-scale GWAS data, we employed MR (an efficient analytical approach) to investigate the association between constipation and PD. MR offers significant methodological strengths for investigating causal relationships, particularly in complex scenarios like the bidirectional link between constipation and PD. It minimizes confounding by environmental, lifestyle, or later-life factors that often bias observational studies. MR isolates the genetic predisposition to constipation, reducing spurious associations caused by shared confounders. It can reduce reverse causation, where disease processes influence observed risk factors which is a critical concern in PD research. Sensitivity analyses detected no evidence of horizontal pleiotropy. Furthermore, our results revealed no association between genetically predicted constipation and PD risk. We observed no consistent pattern linking genetically determined constipation to PD risk. To our knowledge, this represents the first MR study to evaluate the causal relationship between constipation and PD.

Our MR analysis were consistent and does not provide evidence of a causal relationship between constipation and PD, suggesting that the clinically observed association may be biased. However, in this study, we also observed marginal *P*-values (e.g., *P* = .097 for the effect of constipation on PD). Although the *P*-value has not reached the level of significance in the traditional sense, it may still have biological significance. For example, the association between constipation and PD may have been supported in other studies, or the association may be due to some confounding factor that was not controlled for. These findings should be considered exploratory/hypothesis-generating. Consequently, further investigation is required to assess whether residual confounding or methodological biases may have influenced previous observational findings.

This study has several strengths, most notably its MR design, which enables assessment of the causal relationship between constipation and PD while minimizing potential reverse causality and residual confounding.

This study has several limitations. First, although we utilized the largest available GWAS for constipation, our primary analysis included only a limited number of genome-wide significant SNPs (*P* < 5 × 10^‐8^), potentially resulting in weak instrument bias. To address this, we employed a relaxed significance threshold (*P* < 5 × 10^‐6^) to identify additional IVs, all of which had *F*-statistics > 10 to ensure sufficient instrument strength. In our MR analysis of the causal effects of PD on constipation, the calculated OR = 1.003 (95% CI: 0.97–1.04; *P* = .84) indicates that the causal effect of PD (exposure) on constipation (outcome) is very small and not statistically significant. Since the observed OR (1.003) is smaller than the minimum detectable OR (1.04), the analysis may lack sufficient statistical power to detect a significant effect. Possible reasons include a true effect size that is too small, insufficient explanatory power of the IVs. Future studies incorporating larger GWAS datasets are needed to validate our findings. Second, although we found no evidence of horizontal pleiotropy through comprehensive sensitivity analyses, we cannot completely exclude potential pleiotropic effects given the incomplete functional annotation of the selected SNPs. Third, our null findings should be interpreted cautiously, as they may reflect limited statistical power rather than a true absence of association, particularly given potential weak instrument bias and residual confounding inherent even in MR designs. Fourth, the exclusive use of European-ancestry GWAS data limits the generalizability of our results, underscoring the need for future studies in diverse populations. These limitations highlight the importance of subsequent research to confirm our observations.

The complexity of the gut–brain axis makes its role in the relationship between constipation and PD difficult to fully parse, and the function of the gut microbiota is also influenced by a variety of factors, including gut barrier function and immune system status. Together, these factors may influence the occurrence of constipation and PD. Medication use is also an important consideration. Some drugs may affect both intestinal and nervous system function. For example, antipsychotics may cause constipation, while they may also worsen the symptoms of PD. The differences between observational and experimental studies may stem from a variety of factors, including common environmental factors, confounding variables, and the complexity of the gut–brain axis. In addition, nongenetic factors such as lifestyle and medications also play an important role in the development of constipation and PD. Future studies should further explore the specific mechanisms of action of these factors to better understand the relationship between constipation and PD.

## 5. Conclusion

Our study did not find genetic evidence supporting a significant association between constipation and PD in either direction. The robustness of these findings is supported by high-quality genetic instruments, minimal heterogeneity, and comprehensive sensitivity analyses. These results advance our mechanistic understanding of the constipation–PD relationship. Future investigations should examine potential modifying factors and pathophysiological mediators to strengthen clinical translation.

## Author contributions

**Conceptualization:** Huaxing Li.

**Data curation:** Huaxing Li, Weilan Kang.

**Investigation:** Huaxing Li.

**Methodology:** Huaxing Li.

**Project administration:** Huaxing Li.

**Resources:** Huaxing Li.

**Software:** Huaxing Li, Guolan Su, Weihao Huang, Meihui Zhang.

**Supervision:** Guolan Su, Weilan Kang, Weihao Huang, Aiwu Liu, Meihui Zhang.

**Writing – original draft:** Huaxing Li.

**Writing – review & editing:** Guolan Su, Weilan Kang, Weihao Huang, Aiwu Liu.

## Supplementary Material


